# The Evolution of Strategic Timing in Collective-Risk Dilemmas

**DOI:** 10.1371/journal.pone.0066490

**Published:** 2013-06-14

**Authors:** Christian Hilbe, Maria Abou Chakra, Philipp M. Altrock, Arne Traulsen

**Affiliations:** Evolutionary Theory Group, Max-Planck Institute for Evolutionary Biology, Plön, Germany; University of Sheffield, United Kingdom

## Abstract

In collective-risk dilemmas, a group needs to collaborate over time to avoid a catastrophic event. This gives rise to a coordination game with many equilibria, including equilibria where no one contributes, and thus no measures against the catastrophe are taken. In this game, the timing of contributions becomes a strategic variable that allows individuals to interact and influence one another. Herein, we use evolutionary game theory to study the impact of strategic timing on equilibrium selection. Depending on the risk of catastrophe, we identify three characteristic regimes. For low risks, defection is the only equilibrium, whereas high risks promote equilibria with sufficient contributions. Intermediate risks pose the biggest challenge for cooperation. In this risk regime, the option to interact over time is critical; if individuals can contribute over several rounds, then the group has a higher chance to succeed, and the expected welfare increases. This positive effect of timing is of particular importance in larger groups, where successful coordination becomes increasingly difficult.

## Introduction

In joint efforts, coordination problems often arise because some individuals may question the chances of success, or the intentions of the others. In some examples, such as the prevention of climate change [1]–[3] or the management of global economic crises [4], a failure to coordinate on a beneficial equilibrium can endanger the whole group, or implies considerable welfare losses. In all these cases, subjects may try to alleviate the risks of collective action by sending trust-building signals over a period of time. The importance of time as a coordination device that allows individuals to interact and influence one another, has already been noted by Schelling [5]: In the context of multinational conflicts, he argues that “If each party agrees to send a million dollars to the Red Cross on condition the other does, each may be tempted to cheat if the other contributes first, and each one's anticipation of the other's cheating will inhibit agreement. But if the contribution is divided into consecutive small contributions, each can try the other's good faith for a small price.” Thus, the strategic use of time may help to overcome coordination problems that would be hard to settle otherwise.

In order to explore the propensity for such strategic behaviors in humans, Milinski et. al. [6] conducted behavioral experiments for a particular coordination problem, the collective-risk dilemma. In these experiments, each subject was endowed with a fund and then asked, in each of ten consecutive rounds, to donate from this endowment into a common pool. If the group's total contributions after ten rounds reached or surpassed a certain target amount, all group members acquired their individual withheld funds. Otherwise, if the group failed to reach the target, they lost everything with a certain risk probability. In the experiments, a substantial fraction of groups failed to coordinate on a beneficial equilibrium with sufficient contributions, even if the risk of losing everything was as high as 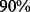. An analysis of the subjects' behavior in these high-risk treatments revealed that there was a significant tendency to procrastinate contributions towards the second half of the game [6]. Such a delay of contributions could be an indicator of individual attempts to free-ride, exploiting the contributions of others. On the other hand, a temptation to wait may also arise if fearful subjects aim to avoid wasted contributions [7].

These observed temporal patterns thus call for a closer examination. However, most previous theoretical investigations for the collective-risk dilemma have neglected the impact of timing on coordination behavior [8]–[13]. These studies considered a one round game and assumed that individuals do not react to the contributions of their co-players over the course of the game. This means that, effectively, timing and thereby strategic behaviors were neglected. An exception is [14], which explicitly followed the setup of the experiments and considered a game with ten rounds. In computer simulations, it was observed that successful players delayed their contributions towards the later stage of the game. However, the focus was on the observable behaviors of the subjects, rather than on the underlying strategies. Moreover, as the game length was fixed to ten rounds, the impact of the duration of the game on cooperation was not analyzed. Herein, we thus add to the previous literature by systematically exploring how time and timing can promote successful coordination.

The impact of time on coordination behavior is probably best explored in the context of the volunteer's dilemma, where a collective good is produced only if there is a volunteer who provides it [15], [16]. For this game, Weesie [17] has found that the inclusion of time greatly enhances coordination and increases the individual probability to volunteer. Moreover, in the asymmetric case where players have different costs of volunteering, time helps to select the optimal volunteer as the player with the lowest costs volunteers without delay [17]. However, there is a subtle difference between coordination in the volunteer's dilemma and in the collective-risk dilemma: even in the symmetric volunteer's dilemma, where players are *ex ante* indistinguishable, the *ex post* payoffs are typically asymmetric – as it takes only one player to take on the burden of volunteering. In contrast, the collective-risk dilemma allows for pure symmetric equilibria, where all players contribute equally to reach the target. Thus, in the collective-risk dilemma the question is not which of the players gives in first, but rather when and to which extent each player contributes.

To address these questions we employ evolutionary game theory [18]–[21]. This allows us to study the dynamics of contributions without presuming that individuals are fully rational (or that they are aware of their co-players' rationality), as for example in [22]. In the following, we thus develop an evolutionary model to show that time has a two-fold effect in the collective-risk dilemma: on the one hand, it facilitates coordination, but on the other hand it leads subjects to delay their contributions as long as possible.

## Model

We consider a collective-risk dilemma played among 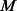 individuals. In each of the 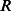 rounds, the players have to decide individually how much of their initial endowment 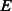 they want to contribute into a common pool. As in the experiments of Milinski et. al. [6], we assume that an individual is limited to a maximum contribution of 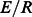 per round, such that a player contributing the maximum amount in each round expends the full endowment 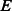. If the group collectively succeeds in investing a target sum 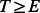 by the end of the game, then each player 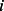 keeps the retained portion of the endowment 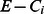, where 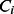 denotes the player's total contributions over the 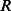 rounds. However, if they collectively fail to reach the target, then all the players lose everything with some exogenous probability 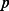. Thus, player 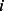 obtains an average payoff of 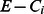 when the target is reached and 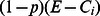 when the target is missed. Overall, the individuals in such a game face a social dilemma: while everyone benefits from reaching the target, players are tempted to suppress their individual contributions.

We model the strategies in such a collective-risk dilemma as contingent rules: when deciding how much to contribute in a given round, players take into account how much their co-players contributed previously. This allows individuals to apply strategies such as Schelling's rule and to contribute an equal amount of 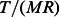 in each round, provided that their peers do the same. Inconveniently, as the number of rounds or the number of players increases, the possible number of contingent strategies increases exponentially. Moreover, collective risk dilemmas have a large set of Nash equilibria: any state in which the group members retain their endowment, or in which they meet the target exactly such that individual contributions do not exceed the expected loss upon failure, 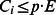, constitutes an equilibrium. To see this, we first note that when the target is exactly met there is no benefit of a further increase of contributions. On the other hand if a player unilaterally decides to cut down her contributions, then the target is missed and the player's payoff is at most 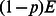, which is below the coordination payoff 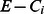 if the risk 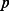 is sufficiently high, 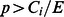. Thus, any outcome where the target is exactly met and where no individual contributes more than 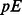 constitutes a Nash equilibrium, even if the costs are distributed unfairly.

To cope with the complexity due to the large number of strategies and possible equilibria, we will study such large-scale collective-risk dilemmas by performing extensive individual-based simulations. However, to provide a basic intuition, we first investigate the role of conditional strategies and timing in a simplified collective-risk dilemma between two players.

### Analysis of a simplified collective-risk dilemma between two players

To illustrate the importance of time as a coordination device in collective-risk dilemmas, let us first explore the baseline case where players are not able to interact over multiple rounds. In such a case, only unconditional strategies are available, such as being a defector (who does not contribute, 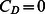), a fair-sharer (someone who contributes a proportional share of the target, 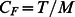), or an altruist (contributing the full endowment, 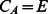). In the simplest case of a pairwise game where the target is equal to one player's endowment, 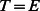, a collective-risk dilemma with these three strategies is represented by the payoff matrix:
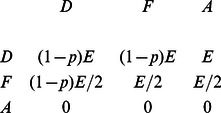
(1)


**Figure 1:**
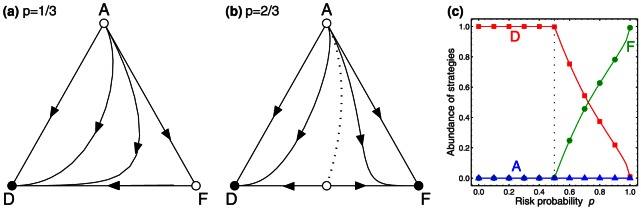
Replicator dynamics of the simplified collective-risk dilemma without timing. For three strategies, the state space takes the form of a triangle, the simplex 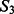. The corners of this triangle correspond to homogeneous populations, where all individuals use the same strategy, whereas points in the interior correspond to mixed populations. (a) If the risk probability 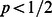, then all interior initial populations eventually converge to a population of defectors. (b) For 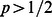 a bistable situation emerges: If the initial frequency of fair sharers is sufficiently high, then the subjects learn to coordinate on the beneficial fair share equilibrium. (c) The fraction of initial populations that converge towards the fair share equilibrium increases with 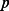, reaching 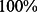 in the limit of full risk. For this graph, we have simulated the replicator dynamics for 20,000 randomly chosen initial populations.

In this game, irrespective of the strategy of their co-player, altruists end up with a payoff of zero. Therefore, altruism is a dominated strategy and we may expect that altruistic acts occur at very low frequencies, reducing the collective-risk dilemma to a game between defectors and fair sharers. Individuals strictly prefer defection for all 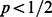, as the expected loss for missing the target 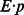 is below the fair share contribution 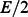. This prediction is confirmed by replicator dynamics [19], [23], see Figure 1a: irrespective of the initial distribution of strategies in the population, individuals learn to stop contributing. This qualitative behavior changes as the risk of collective loss exceeds 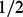. In this case, there are three possible Nash equilibria: all players withholding their contributions, all individuals doing their fair share, and a mixed population of defectors and fair-sharers. In this mixed equilibrium, the fraction of defectors is given by
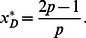
(2)


However, since the mixed equilibrium is not evolutionary stable (Figure 1b), the dynamics either leads to a homogeneous population of defectors or to a homogeneous population of fair sharers. Which of these two possible outcomes is reached, depends on the initial behavior of the individuals: populations with a sufficient initial number of fair sharers eventually succeed in coordinating on the beneficial fair-share equilibrium, whereas populations mostly consisting of defectors end up in the detrimental equilibrium. In general, it depends on the risk of collective loss, whether or not a given initial population succeeds to coordinate on the fair-share equilibrium. To estimate the basins of attraction of each equilibrium, we have recorded the results of the evolutionary dynamics for different randomly chosen initial populations (Figure 1c). According to these simulations, an increasing risk of collective loss stimulates attempts to reach the fair-share equilibrium. Nevertheless, even for high risk values, a substantial proportion of initial populations fails to coordinate on the beneficial equilibrium. For instance, even for 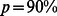, roughly a quarter of all initial states lead to a non-cooperative population of defectors. Increasing the risk of collective loss has therefore a two-fold effect on the achieved welfare: on the one hand, a higher 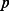 decreases the expected payoff if the target is missed, but on the other hand, high values of 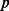 make it more likely that the players cooperate. As a consequence, intermediate, and not high values of 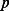 represent the worst-case scenario for the average payoffs (Figure 1c).

To investigate the impact of time, let us now consider a collective-risk dilemma with two rounds. Again, we assume that each agent has an initial endowment 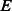, and that each agent can either contribute 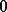 or 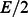 to the common pool in each round. The target is reached if total contributions sum up to a player's endowment, that is 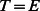. Obviously, this setting allows more than the previous three strategies of defectors, fair sharers, and altruists, as in the two-round game players may condition their behavior in the second round on their co-player's contribution in the first round. We can write the players' strategies as a 3-tuple 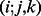 with 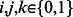. The first variable 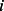 determines whether the player cooperates in the first round: If 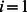, then this player contributes 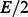 to the common pool, whereas for 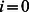, the player does not contribute. The second variable 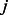 determines whether the focal player cooperates in the second round, given that the opponent cooperated in the first round, whereas the third variable 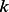 corresponds to the focal player's action in the second round, given that the opponent did not cooperate in the first round.

Therefore, this pairwise collective-risk dilemma allows eight possible strategies, which include the previous three strategies of the game without timing: For example, the strategy 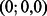 corresponds to a defector who does not contribute to the common pool, whereas players with strategy 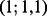 are altruists who contribute their full endowment, independent of the opponent's contribution behavior. The two strategies 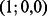 and 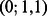 are fair sharers, unconditionally contributing half of their endowment, either in the first period or in the second period, respectively. However, the collective-risk dilemma with timing allows additional strategies of interest: For instance, one may interpret a player with strategy (0;1,0) as a conditional cooperator, who is cooperative in the second round, given that the co-player was cooperative in the first round. In contrast, a player using 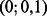 applies a wait & see strategy, by awaiting the first round and by cooperating in the second round if there were no contributions in the first round. We can summarize the eight possible strategies' payoffs in a matrix:
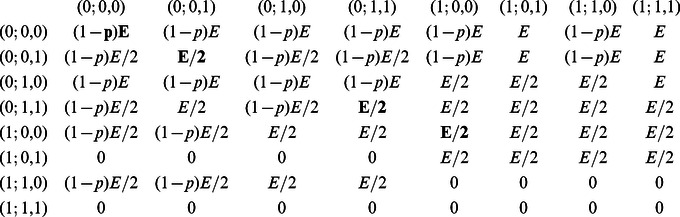
(3)


**Figure 2:**
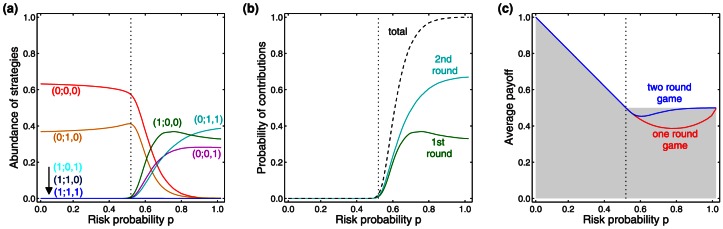
Replicator dynamics of the collective-risk dilemma with timing. a) Monte-Carlo simulations for 20,000 randomly chosen initial populations confirm that individuals are most likely to adopt the defector's strategy if 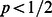, whereas subjects tend to use cooperative strategies for higher risk values. (b) An analysis of the timing of contributions reveals that for high risk values, individuals tend to make their contributions in the second rather than in the first round. (c) Average payoffs in the game with timing are above the payoffs in the game without timing (the grey shaded area represents the set of all possible average payoffs).

Similar to the case without timing, the defector's strategy 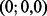 leads to the highest payoff for all risk values 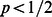. As the risk of collective loss exceeds 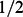, there are three additional pure Nash equilibria, two fair-share strategies 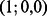 and 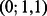 and the wait & see strategy 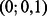. To estimate the robustness of these equilibria, we have again performed simulations with randomly chosen initial populations (see Figure 2a). As expected, defection is the most abundant strategy for low values of 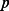. However, for 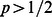, the three cooperative equilibrium strategies 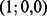, 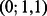 and 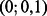 are soon applied by a substantial share of initial populations, leading to complete coordination on a beneficial equilibrium with sufficient contributions as 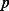 approaches one. Remarkably, for a risk of collective loss of 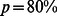, more than 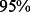 of all initial populations learn to coordinate on an equilibrium with sufficient contributions in this game with timing, while only 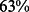 reach the target in the game without timing. The opportunity to interact and influence one another thus indeed proves as a powerful means to reach cooperation in the collective-risk dilemma.

To analyze the timing of contributions, we recorded the fraction of players who contribute in the first and in the second round, respectively (Figure 2b). Depending on the risk of collective loss, one can roughly distinguish three different parameter regions: for 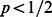, contributions are are neither made in the first nor in the second round, whereas in the interval 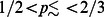, the fraction of contributions increases considerably in both rounds. In this region of intermediate risk, early contributors with strategy 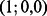 make up the majority of the population (Figure 2a), as they benefit from the presence of conditional cooperators 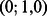. As 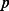 exceeds approximately 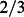, individuals tend to delay their contributions towards the second round. In the limit of full risk, 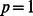, contributions in the second round are twice as likely as early contributions, which is in line with the experimental observation that subjects tend to procrastinate their contributions towards the second half of the game [6].

The positive effect of time on coordination is reflected in the achieved average payoff (Figure 2c); especially for moderate risk values, the two-round game results in substantially higher payoffs than the one-round game. In particular, the minimum payoff increases by more than 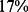 if individuals have the option to interact over time. Again, this minimum payoff is not attained at maximum risk, 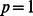, but rather at an intermediate risk value.

### Collective-risk-dilemmas with multiple players and multiple rounds

Real-world coordination problems often involve a large group of individuals and multiple interactions over time. It is therefore natural to explore these more general cases. However, games with multiple players and multiple rounds are considerably more complex, and the size of the payoff matrix increases exponentially in both variables. To investigate such large-scale collective-risk dilemmas, we have performed extensive individual-based simulations. Simulations were conducted using the same setup as in [14], which allows for a comparison with previous work. This setup is similar to the two-round case: each of the 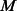 individuals has an initial endowment 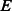 and may contribute at maximum 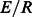 per round to the collective pool, in order to reach the group target 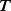, which is set to 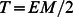 (i.e., the target is reached if all players give their half endowment). For the multi-round case, however, we assume that individuals base their decisions on the collective pool so far, rather than on co-players' individual decisions. That is, for every round 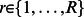 a player defines an individual threshold 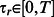 on the total contributions up to round 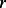. A player's strategy is then a set 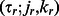, such that the player contributes an amount 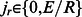 if the common pool satisfies the threshold 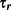, whereas the player contributes 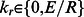 if the individual threshold is not satisfied.

To model the evolutionary dynamics, we use a mutation-selection process in a population of finite size 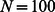. In each generation, individuals participate in several collective-risk dilemmas. Thereafter, the individuals' fitness is calculated as an exponential function of their payoffs, 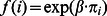, where the strength of selection parameter 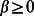 measures the importance of a player's payoff for its fitness. Individuals are then selected in proportion to their fitness to give rise to the next generation [24], [25]. Offspring inherits the strategy of the parent with probability 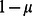; with the remaining probability 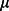 a player explores a randomly chosen new strategy. In case of such a mutation event, we assume that changes in the thresholds and in the investments of each round occur independently, and that changes in the thresholds are normally distributed around a mean of 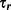 with a variance of 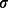. We use this evolutionary game setup to explore the impact of group size, 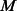, and round number, 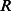, on coordination in collective-risk dilemmas.

**Figure 3:**
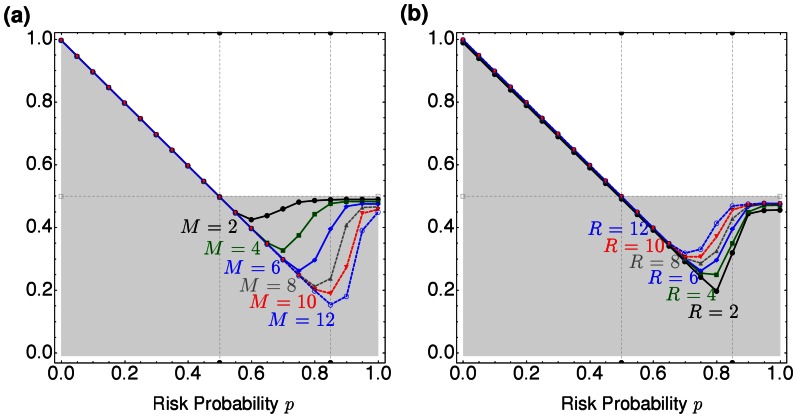
Simulations of the evolutionary dynamics for the collective-risk dilemma with multiple players and multiple rounds. Each graph depicts the average payoff for various 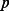, measured in fractions of the initial endowment (the grey shaded area represents the set of all possible average payoffs). (a) A collective-risk game with 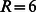 rounds and varying group size, (b) a collective-risk game with 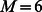 players and varying round number (averages over 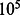 generations, number of games per generation 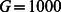, mutation rate 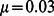, and the standard deviation for mutations in the thresholds 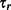 is set to 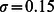).

As one may expect, group size has no effect for low risk values, 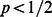, where withholding contributions is a weakly dominant strategy (see Figure 3a). However, for 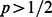, small groups obtain higher payoffs, due to the higher probability to coordinate on a beneficial equilibrium with sufficient contributions. For larger groups, we observe a diffusion of responsibility [26], and it takes higher risks to motivate players to join the collaborative effort. For example, for a risk of collective loss 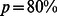, groups of 12 players typically fail to reach the target (resulting in a low payoff of approximately 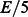), whereas two-player groups almost always reach it (leading to the maximum attainable payoff 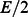). This group effect is quenched when game length increases (see Figure 3b): for 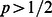, an increase in the number of rounds leads to a higher probability to coordinate on an equilibrium where the target is reached, resulting in a higher average payoff for all players. This positive influence of time is especially pronounced for intermediate risks, such as 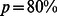. However this quenching did not eliminate the group effect; even if the risk of collective loss approaches one and players have 12 rounds to reach the target, there are still instances of collective failure (resulting in an average payoff below the optimum 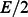). Thus, while the inclusion of time in general facilitates coordination, there is no guarantee that subjects reach the target.

### Timing of Contributions

The previous simulations also allow us to investigate in more detail how individuals time their contributions in games with multiple rounds. If evolution leads to a contribution scheme comparable to Schelling's rule, then we would expect that individuals signal their willingness to contribute already in early rounds, and they would refrain from further contributions as soon as they realize that their co-players do not follow. If applied by all individuals, such a strategy would lead to overall contributions that are evenly distributed over the 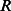 rounds. Alternatively, individuals that are rational could also apply backward induction: for 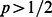, and given that players intend to reach the target, backward induction would suggest that players contribute nothing in the first half of the game, while they would donate the maximum amount 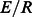 in the second half. In this way, late contributions serve as a self-commitment, which allows individuals to signal credibly that they will not contribute more than their fair share. By not contributing in the beginning, they simply forego any possibility to compensate insufficient contributions, but they ensure that others will either contribute or face collective loss.

**Figure 4:**
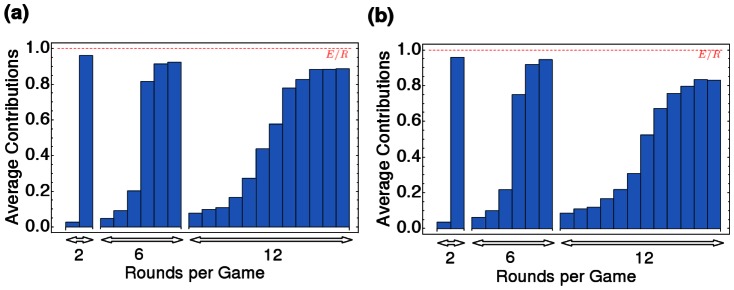
Timing of contributions in the collective-risk dilemma. Simulations of the evolutionary dynamics of a collective-risk game with 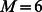, and round number 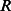 of 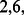 and 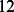 depicting the average contribution per round. We consider two treatments: (a) Possible contributions 0 or 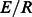. (b) Possible contributions 0, 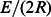, or 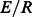. In both treatments we observe delayed contributions, irrespective of the total game length (averages over 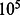 generations, 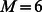, 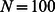, 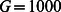, 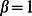, 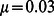, 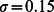).

Our evolutionary simulations suggest that the timing of contributions is somewhere between these two extremes: while average contributions in the last round are typically close to the maximum amount 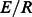, this does not imply that all contributions are shifted towards the second half of the game. Instead, there is always a baseline level of early contributions, independent of the total number of rounds (see Figure 4a). However, the results rather seem to be in line with the backward induction outcome than with evenly distributed contributions over time. This relative abundance of awaiting strategies does not depend on the maximum contribution per round; nor does it depend on the assumption that players only have the binary option of contributing either 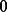 or 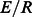 in a given round (see Figure 4b, where subjects could either contribute nothing, 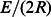 or 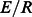).

**Figure 5:**
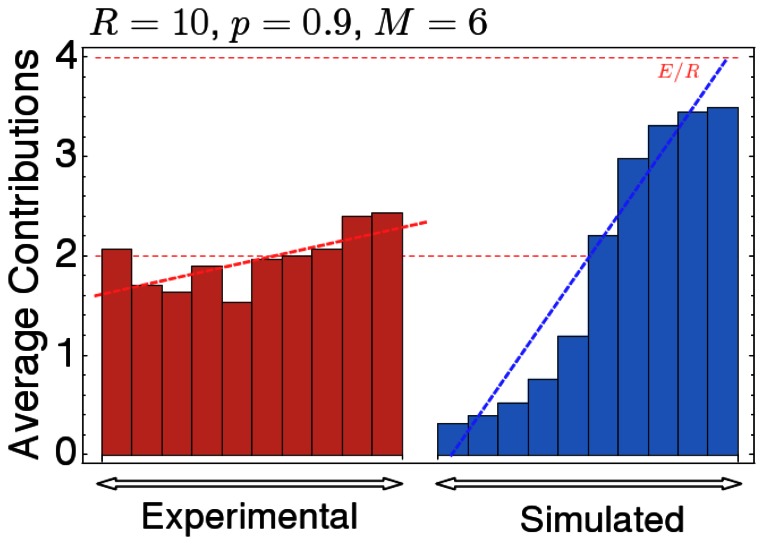
Comparison of the expected timing of contributions according to the simulations with the observed timing in the experiments of Milinski et al.[**6**]. The bold dashed lines show the linear trend, indicating that in the experiments and in our simulations contributions tend to be delayed towards later stages of the game. Parameters were chosen to fit the rules of the experiment, i.e. group size 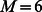, number of rounds 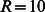, initial endowment 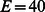, and individuals are allowed to contribute either 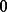, 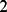 or 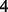 monetary units per round. The other parameters are set to the values in the previous figures.

A significant delay of contributions towards the second half of the game was also observed in the experiments of Milinski et. al. [6]. However, a comparison of the experimental data for a high risk of collective loss, 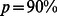 with our results reveals that the evolutionary simulations overestimate the extent of procrastination (see Figure 5). There might be several reasons for this discrepancy: first, in our model individuals only consider the total contributions so far. However, the subjects in experiments may also base their actions on the outcome of the previous round, or on individual behaviors. Unfortunately, reasons behind subjects' actions are still wanting and thus we chose to focus on total contributions. Second, the subjects in the experiments were only allowed to play the game once and thus they did not have the opportunity to learn and adapt their strategies, as assumed in our evolutionary simulations. We would therefore expect that experienced subjects exhibit a behavior that is closer to the backward induction solution, as found in other economic interactions [27]. Third, our evolutionary analysis does not include any psychological motives for contributions, such as loss aversion [28], or framing effects suggesting subjects *should* reach the target by contributing a fair amount each round. The presence of such effects would also explain why subjects contributed a considerable amount of their endowment even in treatments where the risk of collective loss was only 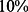, in which case non-contribution would have been the individual and the social optimum. However, if the game is played repeatedly, one might also expect that the impact of these psychological motives decreases [29], and the observed timing of contributions might reveal a stronger tendency to procrastinate.

## Discussion

Examples, such as the prevention of dangerous climate change, or donations to charities, show that many collaborative efforts do not take the form of a one-shot game. Instead, individuals often have the option to await the others' decisions, or to influence others by taking the lead. Here, we have studied how the inclusion of time affects equilibrium selection in a collective-risk dilemma. As a result, we find that time greatly enhances the probability to move towards the efficient equilibrium. This positive effect is of particular importance in larger groups, where successful coordination becomes increasingly difficult [10], [30]. Moreover, we have shown that an increasing risk of an catastrophic event has a two-fold effect on the expected welfare: on the one hand, players have a stronger incentive to coordinate on the beneficial equilibrium, on the other hand it also increases the expected loss upon failure. As a consequence, high risks do not represent the worst-case scenario; rather intermediate risks pose the biggest challenge in collective-risk dilemmas. This result recovers previous observations that severe crises may be actually beneficial for a society, since they increase the probability that necessary measures are adopted [31].

While the inclusion of time facilitates cooperation, it also promotes the evolution of procrastination (which is in line with timing models for public good games, see e.g. [32]). In the extreme case, this may result in strategies that contribute 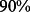 of their share in the very last round (as for example in the two-round game shown in Fig. 4a). Taken together, this may come as a surprise: if players hardly contribute in the early stages of the game anyway, why does the inclusion of these stages increase the probability of successful coordination? It turns out that the fact that most evolutionary trajectories lead towards delayed contributions does not diminish the importance of the early stages. Early contributions help the group to escape from non-cooperative states by motivating conditional cooperators to join in. Once cooperation is established, individuals learn to delay their efforts, because late contributors are less prone to exploitation. Thus, even if early contributions diminish in the long run, they play an important role as a catalyst for cooperation.

Our results thus highlight the importance of time in overcoming coordination problems. Some studies take an opposite view; for example, Drazen and Grilli [31] argue that necessary economic reforms may be delayed if one party attempts to shift the burden of stabilization onto socioeconomic groups that are represented by the other party. In their model, delayed contributions come with a cost, since it prolongs the time spent in an inefficient status quo. In contrast, we have assumed that late and early efforts do not differ in their welfare implications. This may be considered as a limiting case for coordination problems where delayed actions are costly, but where the cost of procrastination is low compared to the stakes in the game (for a model that includes such a cost on late contributions, see [14]). However, *time* should not be taken literally; several instances of collective-risk dilemmas are played over a rather short period (such as efforts to build an emergency sandbag levee by neighbors to protect their community from a flood, [6]). What is crucial, though, is that each player can, directly or indirectly, observe the co-players' actions: it is the flow of information that transforms a one-shot game into a dynamic game, rather than the actual time span (this transformation of the game structure is exactly what is intended when recent donations to charities are publicly announced, instead of made privately, e.g. [33]).

Various generalizations of our model can be addressed. First, we have been considering a homogeneous group, where all individuals are affected equally, and where the quality of contributions does not differ across subjects. Recently, there has been an increasing interest in the impact of inequality [34], [35], investigating the question whether “richer” players would be willing to do a bigger share of the target. While these experiments indeed find that individuals with a high endowment contribute more to the common pool, it was also shown that inequality in general reduces the chance of reaching the target. Second, in our model players could not communicate directly; they could only convey their intentions through their contributions. In contrast, some treatments of Tavoni et. al. [35] allowed subjects to make (non-binding) pledges. Despite being cheap talk, the opportunity to communicate intended contributions increased the success rate dramatically. Typical game-theoretic models have problems to reproduce such an effect of pre-play communication. However, if the game is not considered in isolation, but if players have a reputation to lose (which may affect their performance in future interactions), then modeling the advantages of making pledges seems to be feasible. Herein, we were interested in the human's natural propensity to use time and information to overcome coordination problems, and to motivate others to cooperate. Thus, we have started from a comparably simple model, mimicking the setup of the experiments in [6]. However, we believe that additional communication possibilities will even enhance the group's ability to coordinate on a beneficial equilibrium.
